# Digital Literacy and Its Association With Subjective Health Status and Healthy Lifestyle Behaviors Among Korean Older Adults: Cross-Sectional Study

**DOI:** 10.2196/64974

**Published:** 2025-06-05

**Authors:** Soon Young Lee, Yejin Kim, Bomgyeol Kim, Sang Gyu Lee, Suk-Yong Jang, Tae Hyun Kim

**Affiliations:** 1 Department of Strategic Planning Part, Kangbuk Samsung Hospital, Seoul, Republic of Korea; 2 Department of Public Health, Graduate School, Yonsei University, Seoul, Republic of Korea; 3 Mo-Im Kim Nursing Research Institute, College of Nursing, Yonsei University, Seoul, Republic of Korea; 4 Department of Preventive Medicine, College of Medicine, Yonsei University, Seoul, Republic of Korea; 5 Department of Biohealth Policy Analysis, Graduate School of Transdisciplinary Health Sciences, Yonsei University, Seoul, Republic of Korea; 6 Department of Biohealth Industry Management, Graduate School of Transdisciplinary Health Sciences, Yonsei University, 50-1 Yonsei-ro, Seodaemun-gu, Seoul, 03722, Republic of Korea, 82 0222281521

**Keywords:** digital literacy, healthy lifestyle behaviors, older adults, subjective health status, quality of life

## Abstract

**Background:**

With an aging population driven by advances in medical technology, digital literacy has become essential for improving the quality of life of older adults, enhancing access to health information, and promoting healthy lifestyles. Furthermore, the COVID-19 pandemic may have influenced the subjective health perceptions and healthy lifestyle behaviors of older adults. However, there is limited research exploring the relationship between digital literacy, subjective health perceptions, and healthy lifestyle behaviors in Korea.

**Objective:**

This study aimed to investigate digital literacy’s impact on Korean older adults’ subjective health status and healthy lifestyle behaviors.

**Methods:**

Data of 8664 respondents (aged 65 years and older) from the 2020 National Survey of the Older Koreans were analyzed. Digital literacy was measured based on the use of IT devices (ITDs), difficulty using online information, and inconvenience of ITDs. Statistical analyses, such as the Rao-Scott chi-square test, Wilcoxon rank sum test, and multiple regression analysis, were conducted.

**Results:**

Respondents with above-average ITD use (adjusted odds ratio [aOR] 1.73, 95% CI 1.50‐1.99) and less difficulty using online information (aOR 1.41, 95% CI 1.24‐1.61) had higher odds of perceiving themselves as healthy. Conversely, high difficulty using ITDs was associated with lower odds of respondents perceiving themselves as healthy (aOR 0.84, 95% CI 0.82‐0.87). Furthermore, high ITD use predicted engagement in healthy lifestyle behaviors (aOR 1.51, 95% CI 1.33‐1.72), whereas high difficulty using ITDs predicted lower odds of engagement (aOR 0.94, 95% CI 0.92‐0.97). In contrast, there was no difference in the odds of engaging in healthy lifestyle behaviors regardless of difficulty using online information (aOR 1.03, 95% CI 0.92‐1.15).

**Conclusions:**

This study underscores the significant association between digital literacy and improved health outcomes among older adults. Promotion of digital literacy and relevant policies is essential to help older adults effectively obtain health information online, thereby improving their quality of life and overall health.

## Introduction

Advances in medical technology have increased life expectancy, thereby contributing to the growth of an aging population worldwide [1]. South Korea became an aging society in 2017, and it is predicted to transition to a super-aged society by 2025 [2]. Old age is characterized by a general decline in physical and mental functioning, leading to a range of health issues, including senile and chronic degenerative diseases and impaired daily functioning [3]. Prevention, treatment, and management of diseases are necessary to improve of older adults’ health care and quality of life. In addition, health information and proactive responses for health care are important [4]. Particularly, the first generation of Korean “baby boomers” (born between 1955 and 1972), specifically those born in 1955, has been incorporated into the older adult population since 2020. Thus, this is a crucial time for discussion on older adults’ health care.

Generally, older adults obtain health information from health care providers, friends and relatives, television, newspapers, radio, and other mass media to maintain their health [5]. However, rapid technological developments have increased the opportunities for acquiring health information and news on the internet, leading to digital literacy’s increased importance [6-8]. Digital technology initially spread among young people, men, and highly educated people and is gradually spreading to older adults, women, and less educated people. The aging trend and the rapid development of new media technology suggest that older adults will have to use digital technology more in the future [9]. Digital literacy comprises the ability to understand the information generated on a digital device and implement it accordingly [10]. In an information society, digital literacy’s importance is expanding to all areas of life as a key factor in improving quality of life [11,12].

The manner in which older adults perceive their health informs how they relate to society. In addition, their subjective well-being is intimately linked to their perceived health status [13]. Meanwhile, health concerns can be a primary motivation for searching health information online [14]. Specifically, the use of the internet promotes the health of older adults by improving access to health information, encouraging healthy lifestyles, and enhancing social interaction [15]. While older adults recognize the effectiveness and usefulness of using health information through digital media such as the internet for health care, they experience challenges when actually attempting to obtain health information online [16,17]. According to a 2022 report on the digital divide, vulnerable groups such as people with disabilities, low-income individuals, farmers, and older adults have markedly lower digital use capabilities, with the latter experiencing the highest level of information vulnerability [18].

The growing importance of digital literacy for older adults has prompted a variety of studies. However, most Korean research is survey-based or restricted to specific geographic regions or disease populations, focusing primarily on health knowledge, mental health aspects such as depression and self-efficacy, or quality of life [19-22]. While subjective health perceptions and healthy lifestyle behaviors of older adults may have been influenced by COVID-19 pandemic, these studies were conducted before the pandemic [23-25]. Furthermore, the rapid progress of aging and the advancement of IT, along with gaps in the acquisition of information, unchecked spread of health information, and dissemination of unverified information, have created growing concerns among the older adult population vis-à-vis the lack of reliable information [26-28].

This study seeks to examine 2 key hypotheses: (1) older adults with higher levels of digital literacy are more likely to perceive themselves as healthy and (2) higher levels of digital literacy among older adults are associated with a greater likelihood of engaging in health-promoting behaviors. Using a nationally representative sample, we aim to investigate the association between different aspects of digital literacy and older adults’ subjective health status and healthy lifestyle behaviors.

## Methods

### Data Source and Study Population

This cross-sectional study used data from the 2020 National Survey of the Older Koreans (NSOK), conducted by the Ministry of Health and Welfare and the Korea Institute for Health and Social Affairs (KIHASA). The NSOK is a survey conducted every 3 years since 2008, in accordance with Article 5 of the Welfare of Senior Citizens Act. It systematically collects data from more than 10,000 older adults aged 65 years and older who are living in ordinary residences across 17 cities and provinces nationwide. Trained surveyors carry out the survey in 969 districts selected based on regional representation. They survey all households with older residents, considering factors such as area of residence, age, gender, education, and marital status [29]. This survey aimed to identify the living conditions, characteristics, and needs of older adults to develop welfare policies to improve their quality of life. In addition, it sought to develop policies in response to Korea’s aging society by examining changes in the characteristics of older adults through the accumulation of time-series data [29].

NSOK calculates weights in 3 steps: using design weights, nonresponse-adjusted weights, and poststratification weights. First, the design weights are directly derived from the sampling design, using a 2-stage cluster sampling method. This method involves probability proportional to size (PPS) sampling, where sample areas are selected based on the number of households in each area, followed by the selection of households within each sampled area. Nonresponse adjustment is conducted by upweighting responding households using the reciprocal of the response rate. Final weights are calculated by applying a raking ratio method, and respondent weights, adjusted for design and nonresponse weights, are poststratified to match the distribution of older adults in the population, thereby enhancing the accuracy of estimates. The 2020 NSOK used data from the 2019 Census, the most recent available at the time of survey completion, to calculate final weights by applying population data for older adults by gender, age group (65‐74 years or 75 years and older), and household size (1-person household, 2+ persons) across the 17 metropolitan areas [21].

The study excluded 1256 of the 10,097 respondents who answered “don’t know” or “not applicable” to the digital literacy survey. In addition, if the respondent has a physical or mental illness, the cohabitant or a close representative of the study subject responds on their behalf. In this case, only the objective information of the older adults was surveyed, and the main focus of this study was not surveyed [28]. Therefore, the results were generalized to older Koreans by excluding the 177 respondents who responded on behalf of their cohabitant. These exclusions ensured the objectivity of the health indicator responses, as the survey focused on the subjective judgment of the original respondents [29]. Finally, the study included 8664 participants.

### Ethical Considerations

The Institutional Review Board (IRB) of the Yonsei Medical Center approved a consent exemption for this study (No. 4-2023-0361). The 2020 Elderly Survey used in this study was anonymized by the data provider, KIHASA, and the tools and processes used in the survey were approved by the IRB of KIHASA (No. 2020‐36).

### Measurements

#### Subjective Health Status and Healthy Lifestyle Behaviors

The dependent variables of this study included subjective health status and healthy lifestyle behaviors. Subjective health status has been widely used in various studies as a valid proxy for actual health status, given its reliable predictive value for health outcomes [30-32]. It was categorized into good (very healthy and healthy) and poor (fair, poor, and very poor). Healthy lifestyle behaviors were defined as smoking cessation, decreased alcohol consumption, and exercise [33]. Smoking cessation was defined as having quit smoking, decreased alcohol consumption was defined as not drinking at all in the previous year or drinking less than twice a week, and exercise was defined as answering “yes” to the question, “Do you usually exercise for at least 10 minutes on a consistent basis?”

#### Digital Literacy

This study’s main variable was digital literacy, which was divided into 3 subcategories. It was scored based on the degree of use of IT devices (ITDs), difficulty using information provided online, and inconvenience of ITD usage.

For the item on the degree of ITD usage, 1 point was assigned to each of the activities in Textbox 1, which involved using a PC, mobile phone, or tablet. The sample was divided into 2 groups according to the respondent’s score, namely above average (3 points or more) and below average (3 points or less).

For the item on the difficulty of using information provided online, the degree of difficulty in using information or services required for daily life via the internet was categorized into “difficult” and “not difficult.” The inconvenience of ITD usage was assessed considering tasks such as making online reservations, conducting purchases using machines such as kiosks and automated teller machines, and shopping at stores that only accept cards. For each task, the level of inconvenience was scored on a scale from 0 (“not at all inconvenient”) to 2 (“inconvenient”). Finally, the overall level of inconvenience of using ITDs was rated on a scale from 0 (“not at all inconvenient”) to 8 (“inconvenient”). A Cronbach α of 0.88 was identified for ITD usage, and 0.80 for inconvenience of using ITDs.

Textbox 1. **Activities that involved using a PC, mobile phone, or tablet.**

- Receiving messages.
- Sending messages.
- Searching for and viewing information.
- Taking photos and videos.
- Listening to music.
- Playing games.
- Watching videos.
- Using social media.
- Using e-commerce platforms.
- Using online financial transactions.
- Searching for and installing applications.
- Other activities.

#### Covariates

The covariates included sociodemographic characteristics and health-related factors. Sociodemographic characteristics included gender, age, residence, educational level, household types, and household income. Age was classified into five 5-year intervals (65‐69, 70‐74, 75‐79, 80‐84, and ≥85 years old). Residence was classified into “rural (eup/myeon)” and “urban (dong).” Educational status was classified into “elementary school or less,” “middle and high school,” and “university and above.” Household type was classified into “single,” “married couple,” and “cohabitant (nonspouse).” Household income encompassed all household members’ earned, business, property, and transfer incomes. This comprised the annualized gross household income for the most recent year (July 1, 2019, to June 30, 2020), converted to a monthly basis and divided into quartiles.

Health-related factors included the number of chronic diseases respondents had. Chronic diseases included conditions that lasted for more than 3 months and had been diagnosed by a doctor. These were categorized as “none,” “1‐2 chronic diseases,” and “3 or more chronic diseases,” encompassing 32 conditions.

### Statistical Analysis

A frequency analysis was conducted to identify the participants’ general characteristics. The Rao-Scott chi-square test and Wilcoxon rank sum test were conducted to evaluate and compare these characteristics. Multiple logistic regression analysis examined the associations of digital literacy with subjective health status and healthy lifestyle behaviors. In addition, a subgroup analysis was conducted to determine whether gender led to differences in the association of digital literacy with subjective health status and healthy lifestyle behaviors. All analyses were conducted using SAS software (version 9.4; SAS Institute), while statistical significance was set to *P*<.05. All analyses were weighted by NSOK to account for differences in survey completion rates across NSOK’s survey regions.

## Results

The participants’ demographic and socioeconomic characteristics are summarized in Table 1. Of the 8664 study participants, all weighted by region, 55.9% (4649) had above-average scores in ITD usage, while 72.3% (6264) found it difficult to use information available online. The level of inconvenience of ITD usage was 4.2 out of 8.

Participants were mostly female (n=5077, 55.7%), aged 65‐69 years (n=3282, 35.8%), urban residents (n=6256, 76.1%), educated up to middle and high school (n=4571, 54.4%), married (n=4502, 59.7%), in household income level 4 (n=2169, 29.1%), and had 1‐2 chronic diseases (n=4936, 56.6%).

**Table 1.** Distribution of demographic and socioeconomic characteristics.

Variable	Total, n (%)^a^		Subjective health status					Healthy lifestyle behaviors				
			Healthy, n (%)^a^		Nonhealthy, n (%)^a^		*P* value^b^	Engagement, n (%)^a^		Nonengagement, n (%)^a^		*P* value^b^
Total	8664 (100)		4471 (51.1)		4193 (48.9)			3945 (46.9)		4719 (53.1)		
Degree of ITD^c^ usage							<.001					<.001
Above average	4649 (55.9)		3038 (64.8)		1611 (35.2)			2329 (51.8)		2320 (48.2)		
Below average	4015 (44.1)		1433 (33.8)		2582 (66.2)			1616 (40.7)		2399 (59.3)		
Using information provided online							<.001					.05
Difficult	6264 (72.3)		2898 (45.6)		3366 (0.5)			2773 (46.1)		3491 (53.9)		
Nondifficult	2400 (27.7)		1573 (65.6)		827 (0.3)			1172 (48.9)		1228 (51.1)		
Inconvenience of ITD usage (score), mean (SD)	4.2 (2)		3.7 (1.9)		4.8 (2)		<.001	4.1 (2)		4.4 (2)		<.001
Gender							<.001					<.001
Male	3587 (44.3)		2097 (57)		1490 (43)			1427 (41.4)		2160 (58.6)		
Female	5077 (55.7)		2374 (46.5)		2703 (53.5)			2518 (51.3)		2559 (48.7)		
Age (years)							<.001					<.001
65‐69	3282 (35.8)		2235 (69.3)		1047 (30.7)			1567 (48.2)		1715 (51.8)		
70‐74	2193 (23.9)		1147 (53.9)		1046 (46.1)			1049 (48.6)		1144 (51.4)		
75‐79	1668 (22.4)		640 (38.3)		1028 (61.7)			769 (48.8)		899 (51.2)		
80‐85	1085 (12.9)		322 (27.2)		763 (72.8)			414 (40.9)		671 (59.1)		
≥85	436 (5)		127 (27)		309 (73)			146 (37.2)		290 (62.8)		
Residence							<.001					<.001
Urban (dong)	6256 (76.1)		3361 (52.8)		2895 (47.2)			3055 (50.4)		3201 (49.6)		
Rural (eup/myeon)	2408 (23.9)		1110 (45.9)		1298 (54.1)			890 (35.8)		1518 (64.2)		
Educational status							<.001					<.001
Elementary school or less	3609 (39.1)		1320 (34.3)		2289 (63.4)			1522 (42.5)		2087 (57.5)		
Middle and high school	4571 (54.4)		2790 (60.6)		1781 (39)			2123 (48.2)		2448 (51.8)		
University and above	484 (6.5)		361 (73.3)		123 (25.4)			300 (63)		184 (37)		
Household type							<.001					.01
Single	2693 (19.8)		1117 (39.9)		1576 (60.1)			1160 (43)		1533 (57)		
Married couple	4502 (59.7)		2625 (57)		1877 (43)			2110 (47.8)		2392 (52.2)		
Cohabitant (nonspouse)	1469 (20.5)		729 (44.7)		740 (55.3)			675 (48.3)		794 (51.7)		
Household income							<.001					<.001
Level 1 (lowest)	2158 (20.7)		843 (38.4)		1315 (61.6)			799 (37.3)		1359 (62.7)		
Level 2	2171 (24.2)		965 (42.3)		1206 (57.7)			1044 (49.2)		1127 (50.8)		
Level 3	2166 (26)		1286 (58.2)		880 (41.8)			1020 (46.6)		1146 (53.4)		
Level 4 (highest)	2169 (29.1)		1377 (61.2)		792 (38.8)			1082 (52.1)		1087 (47.9)		
Number of chronic diseases							<.001					<.001
None	1533 (16.9)		1262 (82.1)		271 17.9			730 (48.2)		803 (51.8)		
1‐2	4936 (56.6)		2696 (54.1)		2240 (45.9)			2160 (44.6)		2776 (55.4)		
3 or more	2195 (26.5)		513 (25)		1682 (75)			1055 (51.1)		1140 (48.9)		

a Weighted percentage.

b Rao-Scott chi-squared probability for comparison between (1) healthy and nonhealthy groups, and (2) groups engaging in healthy lifestyle behaviors and those not engaging in healthy lifestyle behaviors.

c ITD: IT device.

Of participants with above-average ITD usage, 64.8% (n=3038) perceived themselves as healthy and 51.8% (n=2329) engaged in healthy lifestyle behaviors. Of participants who reported difficulty using online information, 45.6% (n=2898) perceived themselves as healthy and 46.1% (n=2773) engaged in healthy lifestyle behaviors. Participants who perceived themselves to be healthy exhibited an ITD inconvenience score of 3.7 and those who engaged in healthy lifestyle behaviors exhibited a score of 4.1.

The association of digital literacy with subjective health status and healthy lifestyle behaviors is shown in Table 2. Compared with participants with below-average ITD usage, the odds of participants with above-average ITD usage were 1.73-times more likely to perceive themselves as healthy (adjusted odds ratio [aOR] 1.73, 95% CI 1.50‐1.99, *P*<.001) and 1.51-times more likely to engage in healthy lifestyle behaviors (aOR 1.51, 95% CI 1.33‐1.72, *P*<.001). Participants who did not find it difficult to use information available online had 1.41-times (aOR 1.41, 95% CI 1.24‐1.61, *P*<.001) higher odds of perceiving themselves as healthy, compared with those who found it difficult. Higher ITD difficulty scores were also associated with 0.84-times lower odds of respondents perceiving themselves as healthy (aOR 0.84, 95% CI 0.82‐0.87, *P*<.001) and 0.94-times lower odds of engaging in healthy lifestyle behaviors (aOR 0.94, 95% CI 0.92‐0.97, *P*<.001).

**Table 2.** Digital literacy and its association with subjective health status and healthy lifestyle behaviors.

Variable	Perceived as healthy		Engaging in healthy lifestyle behaviors	
	aOR^a^^,^^b^ (95% CI)	*P* value	aOR^a^ (95% CI)	*P* value
Degree of ITD^c^ usage				
Below average	Ref.^d^	—^e^	Ref.	—
Above average	1.73 (1.50-1.99)	<.001	1.51 (1.33-1.72)	<.001
Using information provided online				
Difficult	Ref.	—	Ref.	—
Nondifficult	1.41 (1.24-1.61)	<.001	1.03 (0.92-1.16)	.59
Inconvenience of ITD usage (score)	0.84 (0.82-0.87)	<.001	0.94 (0.92-0.97)	<.001

a Adjusted for covariates including gender, age, residence, educational status, household type, household income, and number of chronic diseases.

b aOR: adjusted odds ratio.

c ITD: IT device.

d Ref.: reference.

e Not applicable.

The association of digital literacy with subjective health status and healthy lifestyle behaviors by gender is shown in Tables3  4. Both men and women with a higher than average degree of ITD usage were more likely to perceive themselves as healthy than those with an average or lower degree of ITD usage, and women in particular were nearly twice as likely (male aOR 1.64, 95% CI 1.34‐1.99, *P*<.001; female aOR 1.91, 95% CI 1.60‐2.27, *P*<.001). Similarly, people who did not experience any difficulties in using information provided online were more likely to perceive themselves as healthy than those who did experience difficulties, but this was more likely to be the case for men (male aOR 1.60, 95% CI 1.32‐1.94, *P*<.001; female aOR 1.27, 95% CI 1.06‐1.51, *P*=.01). Women with a higher degree of ITD usage were 1.54 times more likely to engage in healthy lifestyle behaviors than women with a lower degree of ITD usage, and this was slightly higher than for men (male aOR 1.47, 95% CI 1.20‐1.81 *P*<.001; female aOR 1.54, 95% CI 1.30‐1.82, *P*<.001). The less comfortable they were with using ITD, the less likely they were to perceive themselves as healthy (male aOR 0.84, 95% CI 0.80‐0.88, *P*<.001; female aOR 0.85, 95% CI 0.81‐0.88, *P*<.001) and to practice healthy lifestyles (male aOR 0.95, 95% CI 0.90‐0.99, *P*=.02; female aOR 0.94, 95% CI 0.90‐0.97, *P*<.001), with similar results for men and women.

**Table 3.** Digital literacy and its association with subjective health status by gender.

Variable	Perceived as healthy			
	Male		Female	
	aOR^a^^,^^b^ (95% CI)	*P* value	aOR^a^ (95% CI)	*P* value
Degree of ITD^c^ usage				
Below average	Ref.^d^	—^e^	Ref.	—
Above average	1.64 (1.34-1.99)	<.001	1.91 (1.60-2.27)	<.001
Using information provided online				
Difficult	Ref.	—	Ref.	—
Nondifficult	1.60 (1.32-1.94)	<.001	1.27 (1.06-1.51)	.01
Inconvenience of ITD usage (score)	0.84 (0.80-0.88)	<.001	0.85 (0.81-0.88)	<.001

a Adjusted for covariates including age, residence, educational status, household type, household income, and number of chronic diseases.

b aOR: adjusted odds ratio.

c ITD: IT device.

d Ref.: reference.

e Not applicable.

**Table 4.** Digital literacy and its association with healthy lifestyle behaviors by gender.

Variable	Engaging in healthy lifestyle behaviors			
	Male		Female	
	aOR^a^^,^^b^ (95% CI)	*P* value	aOR^a^ (95% CI)	*P* value
Degree of ITD^c^ usage				
Below average	Ref.^d^	—^e^	Ref.	—
Above average	1.47 (1.20-1.81)	<.001	1.54 (1.30-1.82)	<.001
Using information provided online				
Difficult	Ref.	—	Ref.	—
Nondifficult	1.08 (0.91-1.29)	.38	1.00 (0.85-1.18)	.96
Inconvenience of ITD usage (score)	0.95 (0.90-0.99)	.02	0.94 (0.90-0.97)	<.001

a Adjusted for covariates including age, residence, educational status, household type, household income, and number of chronic diseases.

b aOR: adjusted odds ratio.

c ITD: IT device.

d Ref: reference.

e Not applicable.

## Discussion

### Principal Findings and Comparison With Previous Work

This study examined the association between older people’s digital literacy and their subjective health status and healthy lifestyle behaviors. Research has shown that older people with a high level of digital literacy, those who actively use information devices, and those who have easy access to online information are more likely to feel healthy and maintain healthy lifestyle behaviors. Conversely, those who experience difficulty accessing information online and are uncomfortable using information devices are less likely to engage in healthy lifestyle behaviors.

This study’s findings are consistent with those of previous studies showing that both the use and amount of information affect subjective health status. In addition, whether older people own digital devices, how often they use them, and the extent of their internet activity are likely to affect their digital literacy and health [34-36]. According to a study in the United States, searching for and mastering relevant medical knowledge on the internet is beneficial for older adults with high blood pressure and heart disease, improving their health care and reducing disease rates [37]. In addition, using the internet could improve the health of older individuals by increasing their ability to exercise; for example, internet intervention programs helped them select exercise activities in the areas of endurance, flexibility, strengthening, and balance enhancement, which helped older adults increase their exercise frequency, thereby improving their physical health [38,39]. In China, older people with a high frequency of digital device usage experience a positive impact on their lifestyle, including improvements in diet, sleep, and exercise [15,40].

Empowering older people with a wide range of digital skills, from simple computer use to usage of social networks, emails, and online meetings, could enrich their lives, enhance their cognitive capabilities, and make them more productive and efficient members of society [41]. This could significantly contribute to the improvement of their perceived health status [42]. People who understand health information available online can use it effectively in their daily life to acquire valuable insights regarding their illnesses, increase their involvement as patient in the treatment process, and positively assess their subjective health status [43]. The internet provides a wealth of information, creating and increasing thematic content, especially for groups intimately associated with older adults, which helps maintain intergenerational relationships and promotes connected social capital [44]. With this social capital, older adults with a high level of digital literacy could fulfill their desire for belonging and love, which in turn could enable them to evaluate their health more positively [45].

However, unlike previous studies, this study found no difference in healthy lifestyle behaviors between those with and without difficulty using online information. Older people with high digital literacy have easier access to health information and use it effectively to positively impact their health care. Furthermore, they regularly access the internet for health information [27,35]. A previous study reported that adults aged 50‐60 years who are not in good health access the internet for health care purposes because they want to be actively involved in health care decisions [46]. A few older individuals use emails and social networking sites to promote cancer screening to colleagues [47]. Online mediation, such as effectively managing hemoglobin A_1c_ levels in patients with diabetes through social networking site–based interventions, improved knowledge about the disease and had a positive impact on health behaviors [48,49].

Also in South Korea, Min [50] reported that the use of internet information decreased with age, regardless of physical and environmental accessibility, but this measure of use was comparable with this study’s degree of ITD usage. Previous studies have also highlighted the positive effects of online health information seeking psychological and physical domains, such as increased self-efficacy and improved health behaviors [51,52]. However, these studies were conducted before the COVID-19 pandemic, leaving a gap in understanding health behaviors during and after the pandemic.

The 2020 NSOK survey was conducted during the COVID-19 pandemic—a period marked by reduced outings among older adults due to social distancing policies and concerns about weakened immune systems. This context likely contributed to an overall decline in healthy lifestyle behaviors, particularly exercise, regardless of difficulties in using online information [23-25]. The reliance of Korean older adults on interpersonal sources for health information is deeply rooted in cultural values such as Confucianism and collectivism [53,54]. For health-related information, older adults in South Korea often rely on various sources, including health care professionals, mass media, local health centers, and family members. Particularly, adult children act as intermediaries in accessing and interpreting digital health information. In addition, a strong cultural trust in health care professionals reinforces a preference for direct consultations rather than independent online searches [55-57]. These sources play an important role in supporting health behaviors, even without the use of the internet, and help create an environment where information remains accessible despite low levels of online information use. In addition, older adults may have relied on household members or health center staff to mediate and explain online information, rather than accessing it directly [58-60]. This indirect approach could have resulted in an underestimation of the actual challenges older adults face when navigating online information.

### Limitations

The present study had a few limitations. First, this is a part-sectional study. As we measured the variables at the time of the survey, we could only derive meaning in relation to the association between older adults’ digital literacy and the causal relationship between their subjective health status and health practices. Second, as life expectancy increases, it is possible to divide the older adult population aged 85 years or older into different age groups. However, the NSOK is limited to detailed age-specific analysis. Nevertheless, this study used the latest data from national units, which ensured the representation of South Korea’s older adults. In this study, digital literacy was divided into specific categories such as the degree of ITD usage, the difficulty of accessing online information, and the inconvenience of ITDs. This highlights the importance of linking older people’s digital literacy to their subjective health status and health practices.

### Conclusions

This study expands the existing knowledge on the subject by using a national-scale survey of South Korea’s older adult population. To improve the subjective health and health practices of older adults, digital literacy education and related policies must be promoted. Furthermore, it is important to provide information education, so that older individuals can identify the required information, even among commercial and vague content. Health care and public institutions should provide information, booking systems, guidance, and other services to help older adults make use of these resources. We hope that this study will serve to improve older adults’ quality of life and contribute to their health and welfare.

## Abbreviations

aOR: adjusted odds ratio
IRB: Institutional Review Board
ITD: IT device
KIHASA: Korea Institute for Health and Social Affairs
NSOK: National Survey of the Older Koreans
